# Transformational Leadership, Organizational Resilience, and Team Innovation Performance: A Model for Testing Moderation and Mediation Effects

**DOI:** 10.3390/bs15010010

**Published:** 2024-12-26

**Authors:** Jiayu Yu, Kaibiao Xiang

**Affiliations:** 1 School of International Studies, Peking University, Beijing 100091, China; yujiayu@stu.pku.edu.cn; 2 School of Management, Guizhou University, Guiyang 550025, China

**Keywords:** transformational leadership, organizational resilience, environmental uncertainty, team innovation performance, regression analysis

## Abstract

This study adopts transformational leadership as the research subject and aims to explore the effect of transformational leadership on organizational resilience and team innovation performance: to test the mediating role of organizational resilience between transformational leadership and team innovation performance, as well as to examine the moderating role that environmental uncertainty performs in the process of the mediation. By analyzing the data collected from a questionnaire, this research discovered that transformational leadership has a positive effect on team innovation performance, which is mediated by organizational resilience. This relationship is more significant when environmental uncertainty is considered as a moderator in the mediation process. Ultimately, based on the findings illustrated above, this study provides suggestions to improve team innovation performance by developing transformational leadership capabilities and awareness, enhancing the business environment, boosting organizational resilience, and increasing the resilience of enterprises in the context of environmental uncertainty. The contribution of this research is demonstrated by the provision of a set of scientifically grounded recommendations for the administration of organizations and enterprises in underdeveloped regions of China.

## 1. Introduction

Continuous adaptation to changes and innovation are essential for organizations to sustain a competitive advantage in the contemporary business environment (Teece, 2007; H. F. Lin & Sun, 2024). Transformational leadership constitutes a crucial factor that triggers innovation and creativity within enterprises (H. L. Zhang et al., 2021a). Transformational leaders possess a positive mindset and vision that foster passion and creativity among team members (Waldman & Yammarino, 1999; H. P. Sun & Hansen, 2020), the capacity to undertake risks, as well as the ability to rapidly adjust to change and even take advantage of it (Ma et al., 2024), thereby facilitating innovation within the organization. Nevertheless, organizations frequently encounter a variety of obstacles while attempting to respond to innovation and change. Within this context, the discourse regarding organizational resilience is of paramount significance. Organizational resilience refers to the ability of an organization to adapt, change, and recover in circumstances consisting of stress, challenge, and uncertainty. Resilience encompasses not merely the capacity to adjust within the organization but also to interact with and adapt to the external environment (X. E. Zhang & Teng, 2021). In the process of adapting to changes, organizations with a high level of resilience are able to maintain stability and development, thereby promoting team innovation performance. This result is achieved by promptly responding to the shifting market conditions, technological changes, and competitive pressures (Tao et al., 2024). Team innovation performance is defined as the results achieved by the team in innovative activities, which is crucial to the development and competitiveness of organizations. However, team innovation performance is influenced by various factors, including the leadership style and the resilience of an organization (Yin et al., 2020).

Previous research has explained the factors that influence team innovation performance. Nevertheless, the extent to which transformational leadership impacts the performance of team innovation, with organizational resilience serving as a mediator, in the context of environmental uncertainty has not been thoroughly explored. This study employed a questionnaire to gather information regarding team performance, leadership and employee development, organizational resilience, and shifts in the context of environmental uncertainty among enterprises in Guizhou Province, in China, which is an economically underdeveloped region. The collected data have been analyzed through statistical analytic methods. Based on the collection and analysis of primary sources and data, the relationship between the above-mentioned variables has also been examined. This research aims to provide empirical findings on the leadership and management strategies of enterprises in the Chinese context, which assists enterprises in improving innovation performance and adapting to the uncertain environment through the empirical study of transformational leadership, organizational resilience, and team innovation performance.

## 2. Literature Review

### 2.1. Transformational Leadership

Transformational leadership behavior is a highly prominent theory in contemporary leadership research (Y. Zhang & Hou, 2013). The concept was first introduced in Downton’s (1973) book titled “Rebel Leadership”, which examined how leaders motivate their followers to foster transformation collectively through their compelling personalities and commitments in the context of organizational change. Building upon this foundation, Burns (1978) first presented the notion of transformational leadership in his book “Leadership”, defining transformational leadership as the leader’s ability to enhance the motivation and goals of followers through personal charisma, individualized consideration, and intellectual inspiration, prompting them to achieve more ambitious goals. Furthermore, Chinese scholars further explored the notion of transformational leadership. According to Qi and Zhang (2001), transformational leadership behavior is a process in which leaders motivate their employees and foster ideological and ethical values among them. Based on the four-dimensional structure of transformational leadership proposed by Bass and Avolio (1990), including charisma, inspirational motivation, intellectual stimulation, and individualized consideration, C. P. Li and Shi (2005) further domesticated these four dimensions into moral example, visionary inspiration, charisma, and personalized care for the purpose of better explaining transformational leadership within the Chinese context. To put it in a nutshell, academics share a common perspective on the essence of transformational leadership, which is the capacity of a leader to motivate and inspire team members through personal charisma, intellectual stimulation, and individualized care in order to trigger significant change and innovation among organizations and society.

A number of scholars have developed their own assessment criteria to evaluate the components of transformational leadership. Early research on transformational leadership concentrated mainly on three dimensions: charismatic-inspirational leadership, intellectual stimulation, and individualized consideration. Bass and Avolio (1996) further distinguished “charismatic-inspirational leadership” into two dimensions, namely leadership charisma and inspirational motivation, leading to the development of the four-dimensional framework of transformational leadership along with the corresponding evaluation tool, the Multifactor Leadership Questionnaire (MLQ). The four-dimensional framework of transformational leadership has been widely acknowledged, as well as the MLQ, which emerged as the predominant tool in the evaluation of transformational leadership. Its theoretical significance and accuracy of predictions have also been proven by several empirical studies. However, Chinese business leaders present a unique paternalistic leadership style (Farh et al., 2000). In light of the Chinese cultural context, scholars C. P. Li and Shi (2005) localized the four-dimensional framework of transformational leadership as follows: moral example, visionary motivation, leadership charisma, and personalized consideration, and designed the transformational leadership questionnaire (TLQ), which has been widely embraced.

Organizational resilience is a remarkable characteristic for effectively addressing multiple adversities, allowing teams to resist risks, overcome obstacles, and ultimately accomplish greater development (M. X. Sun et al., 2021). Due to the fact that organizational resilience generates positive consequences and can be enhanced through cultivation and reinforcement, resulting in scholars primarily focusing on its causes (Y. H. Zhu et al., 2019). From a comprehensive viewpoint, leadership performs a crucial role in the establishment and growth of organizational resilience among enterprises (Kuntz et al., 2017). Transformational leadership influences and strengthens organizational resilience by facilitating organizational construction, resource allocation, and development opportunities for structural adjustment and enhancement. As a result, with the influence of transformational leadership, enterprises are better equipped to respond to adversity and make effective modifications during times of crisis, enabling them to bounce back from setbacks and failures rapidly.

### 2.2. Team Innovation Performance

Team innovation refers to the intentional introduction of innovative ideas and their subsequent practical application by a team within the enterprise (Ancona & Caldwell, 1992; Xin & Miao, 2018). Team innovation performance evaluates the level and effectiveness of team innovation (Tatikonda & Rosenthal, 2000; M. Li et al., 2024) and reflects the extent to which the team accomplishes its innovative objectives (Katz & Allen, 1985; Zeng et al., 2016). Innovation entails an interactive process of learning (Y. H. Tang et al., 2020), and team innovation transcends the mere accumulation of individual innovations (Guo et al., 2012).

Previous research has highlighted the importance of transformational leadership in promoting positive organizational outcomes such as knowledge production, innovation, and employee engagement. Scholar Yoo et al. (2020) investigated the impact of transformational leadership on knowledge creation practices in educational settings, emphasizing the mediating role of a supportive learning culture and work engagement. Although transformational leadership has a substantial impact on knowledge creation methods in both countries, leadership has a stronger direct influence on knowledge creation in Korean schools, but a supportive learning culture is more prevalent in US schools. Similarly, Kasımoğlu and Ammari (2020) also conducted a comparative analysis focusing on the impact of transformational leadership on employee creativity in Turkey and Algeria, with the mediating effect of employees’ creative role identity (CRI) and therefore extended the discussion to non-Western settings. This study discovered that, while Turkish managers recognize the efficiency of every component of transformational leadership independently, including idealized influence, inspirational motivation, intellectual stimulation, and individualized consideration on employee creativity, Algerian managers regard CRI as a significant mediator in the relationship between transformational leadership and employee creativity.

Caza et al. (2021) examined the universality of transformational leadership behaviors and their effectiveness in 77 countries. It discovered that transformational leadership is universally perceived similarly across cultures; it also results in similar levels of follower satisfaction, indicating its potential as a universally accessible leadership practice. Farzana and Charoensukmongkol (2024) also highlighted the cross-cultural effectiveness of transformational leadership by the comparative analysis of Bangladesh and Thailand. This study emphasized the moderating role of national culture, specifically power distance, in the relationship between transformational leadership and psychological empowerment. Transformational leadership has a greater positive effect on psychological empowerment in cultures with higher power distances, such as Thailand, and that psychological empowerment mediates the relationship between transformational leadership and employee engagement.

As a typical leadership style, the relationship between transformational leadership and team innovation performance is a topic of controversy. Studies conducted by researchers, including Jung et al. (2003), have proven that transformational leadership has a positive effect on the performance of organizational innovation. Chinese scholars, namely J. Zhang and Wang (2020), agreed with this finding, although they placed greater focus on the process by which transformational leaders improve team innovation performance by enhancing their own work input. Subsequent research challenges this perspective by asserting that there is no substantial correlation between transformational leadership and team innovation performance (S. Y. Zhu et al., 2008). However, the effect between these two variables tends to be achieved through information sharing and knowledge integration (Ke et al., 2009). Furthermore, the correlation between transformational leadership and team innovation performance is influenced by the innovation climate of teams, with a positive correlation in favorable emotional climate and a negative correlation in unfavorable emotional climate (X. Y. Liu & Liu, 2012).

Lovelace et al. (2001) have developed team innovation performance indicators that evaluate four dimensions: the team’s adaptability to change, the number of innovations or new ideas introduced by the team, the team’s overall technical performance, and the innovativeness of the team’s product. These indicators have been cited by a number of scholars, demonstrating their high reliability and cultural adaptability in China.

### 2.3. Organizational Resilience

As discussed above, resilience enables organizations to resist risks, overcome adversity, and even attain accelerated and enhanced development (M. X. Sun et al., 2021). According to Liang and Cao (2021), organizational resilience refers to the ability of organizations to effectively respond, adapt, and recover from changes and uncertainties. Achieving this capability requires the implementation of several strategies and resources, including anticipating potential threats, responding effectively to unexpected events, and learning in the process of problem solving (Hillmann, 2021; Y. Li & Liang, 2024). Organizational resilience entails not merely the ability to survive and recover in the context of adversity but also the capacity to identify and take advantage of new opportunities during a crisis (R. Chen et al., 2021), actively update and redesign its organizational structures and relationships, and ultimately achieve development against the adversity (Munoz & Billsberry, 2024; J. B. Lin & Fan, 2024).

Quantitative analysis constitutes a fundamental component of both theoretical study and practical exploration in the field of business management (C. Chen et al., 2023). Since organizational resilience (OR) has multidimensional and multi-level characteristics (G. H. Wang et al., 2024a), the evaluation of this concept can be divided into various dimensions and categories, each representing a different capability or characteristic of an organization when facing a crisis, consisting of cognitive, behavioral, and situational dimensions, as well as capital, strategic, relational, cultural, and learning resilience (Williams et al., 2021; J. R. Wang & Liu, 2024). Several studies have also quantified organizational resilience through different aspects, such as long-term performance growth and financial volatility (Wu & Feng, 2022); performance growth and financial volatility (Gao et al., 2024).

Although transformational leadership behaviors have a direct impact on team innovation performance, various intermediate transmission mechanisms are likely to occur. Within an uncertain business environment, team resilience constitutes a crucial factor for teams to confront risks, recover efficiently, and even attain development afterwards (B. N. Liu & Zhang, 2023). The construction of organizational resilience is a complex system, in which enhancing it requires positive interactions among various sub-systems (B. J. Zhang & Pei, 2020). In particular, transformational leadership and the heterogeneity of the human resources of the executive team both have a significant positive effect on organizational resilience (M. X. Sun et al., 2021; Hu et al., 2021).

In addition to the direct effect discussed above, the relationship between transformational leadership and organizational resilience is further influenced by the external environment (Zhao et al., 2021). While experiencing socioeconomic uncertainties, visionary motivation encourages employees by strengthening their willingness to respond to external risks collaboratively and engaging in innovative activities (Chi et al., 2023). In the case of weak business performance, strategic transformation has a negative impact on organizational resilience (Zhou et al., 2024). To mitigate this adverse effect, enterprises are encouraged to enhance the positive leadership of their executives (Y. Wang & Cai, 2021; J. Zhang et al., 2021b); develop dual innovation to improve their core capabilities in both technological and market fields (Y. N. Sun & Jiang, 2024); accelerate digital transformation (Q. Wang et al., 2023), intelligent transformation (S. S. Zhang & Dong, 2024), and technological diversification (H. H. Wang et al., 2024b), for the purpose of enhancing organizational resilience.

Organizational resilience is strongly associated with entrepreneurial innovation (Hou et al., 2023; Ye et al., 2022; C. Y. Tang et al., 2023), in which prospective resilience and improvisational resilience have a significant positive effect on technological innovation (J. R. Wang et al., 2024c). A number of studies have explored the mediating role performed by organizational resilience, including a partial mediating role between platform leadership and sustainable innovation capability (Liang et al., 2023); a significant mediating effect between technological innovation and innovation performance; a partial mediating role of internal resilience between reactive cross-boundary search and green technological innovation; and a partial mediating role of external resilience among prospective cross-boundary search and green technology innovation (J. R. Wang & Liu, 2024; J. R. Wang et al., 2024d). In addition, organizational resilience effectively resists the adverse effects of financial mismatches on green innovation.

### 2.4. Environmental Uncertainty

Enterprises operate in a highly competitive, uncertain, and complex environment that requires flexibility and adaptability. This specific environment is defined by rapid market and technological change, severe competition, and shifting consumer needs and preferences, which is identified as “environmental uncertainty” (Eisenhardt, 1989). Duncan (1972) initially introduced the concept of environmental uncertainty, defined by the rate and complexity of changes occurring in the environment. Based on this concept, Milliken (1987) stated that uncertainty in dynamic environments falls into three categories: state uncertainty, effect uncertainty, and response uncertainty. Since the 1990s, research on environmental uncertainty has been further developed. The concept of “environmental dynamism” emphasizes the impact of the pace and unpredictability of environmental change on the organizational structure and strategy of enterprises (Tushman & O’Reilly, 1996). The theory of “dynamic capabilities” further enhances the exploration of environmental uncertainty, suggesting that enterprises in dynamic environments require constantly updating and reshaping their internal resources and capabilities to address changes in the external environment (Teece et al., 1997). In the 2000s, scholars have focused on the perspectives of networks and ecosystems in environmental uncertainty. From these perspectives, enterprises not merely focus on their own innovation capabilities but also on the collaborative innovation capabilities of the entire industrial ecosystem to respond to environmental uncertainty (Adner, 2006). Therefore, it requires the establishment of an adaptive, open, and inclusive culture that promotes both internal innovation and external cooperation in order to gain an advantage in the highly competitive market (Birkinshaw et al., 2016).

As discussed above, the concept of environmental uncertainty has been evolving from the pace and complexity of environmental change to the uncertainty and further to dynamic enterprise capabilities and ecosystem cooperation. In response to environmental uncertainty, modern enterprises are expected to maintain a high degree of flexibility, adaptability, and the capacity to rapidly respond to uncertainties and challenges through continuous innovation and collaboration.

The common approach for measuring environmental uncertainty comprises both subjective and objective approaches. The subjective approach primarily adopts questionnaires to investigate the perception of enterprise managers regarding the uncertainty of the environment, while the objective approach relies on analyzing data from the industry, such as the market growth rate, the pace of technological development, and other indicators (Deng et al., 2021). These two approaches have their own advantages and disadvantages. Both of these approaches possess inherent drawbacks. The subjective approach reflects the genuine perceptions of managers; however, it tends to incorporate personal bias. Conversely, the objective approach is likely to inadequately capture the specific environment in which the enterprise is situated.

Subjective measurement approaches are generally conducted through questionnaires assessing managers’ perceptions of the external environment. For instance, Dess and Beard (1984) designed the Environmental Dynamics Scale, encompassing market change, technological change, and competitive pressure, while Miller and Friesen (1983) formulated a package of criteria measuring the environmental uncertainty of enterprises, which covers the dimensions of market dynamics, technological dynamics, and the dynamics of competition. Scholars have also measured environmental uncertainty through the application of environmental complexity and environmental dynamics, which provide a framework for assessing the environmental uncertainty of enterprises, providing effective approaches to measure these factors in the Chinese context (J. Chen et al., 2022).

Objective measurement approaches assess the uncertainty of the environment primarily by analyzing macroeconomic data and industry statistics. For instance, indicators including market growth rate (Seo et al., 2020), pace of technological innovation (Schilling, 2017), and frequency of policy changes (Dess & Beard, 1984) could be employed to measure the extent of environmental uncertainty. Similarly, Chinese scholar L. B. Liu et al. (2023) discovered that financial, internal control, and governance characteristics are able to influence the uncertainty of the external environment by employing the 2D-UEE model, which provides an innovative approach for the comprehensive measurement of the environmental uncertainty that enterprises experienced. By analyzing these objective data, a more comprehensive understanding of the external environment faced by enterprises can be obtained (Sharfman & Dean, 1991).

In recent years, researchers have explored a hybrid approach that combines subjective and objective approaches in order to compensate for the shortcomings of adopting a single method discussed above. D. Li and Liu (2014) proposed a method for measuring environmental dynamism that combines subjective perceptions and objective data, which enhances the accuracy and comprehensiveness of the measurements through integrating the subjective assessments of enterprise managers and the industry data. Analyzing market and technological changes through database and artificial intelligence techniques constitutes one of the methods to capture environmental uncertainty more precisely (Wamba et al., 2024; Davenport & Ronanki, 2018; C. L. Wang et al., 2015). In addition, social media data and internet search trends have also been considered as proxy variables of environmental uncertainty as a way to improve its measurement accuracy through real-time data analysis (Choi & Varian, 2012; Heredia et al., 2022).

Within an uncertain business climate, the ability of enterprises to maintain a competitive advantage is largely dependent on their organizational resilience and team innovation performance. As an important external factor, environmental uncertainty performs an essential role in moderating the relationship between organizational resilience and team innovation performance. The dynamic capabilities framework proposes that in an unpredictable environment, enterprises need to develop and apply dynamic capabilities to sense, seize, and reconfigure opportunities in order to adapt to and shape market and technological changes. This theoretical framework emphasizes the importance of organizational resilience in maintaining business operations and competitiveness in the context of environmental uncertainty. Since environmental uncertainty increases unpredictability and complexity, enterprises are required to quickly adapt to and take advantage of the market shifts (Teece, 2007). Hence, within environmental uncertainty, organizational resilience has a greater impact on team innovation performance, whereas the effect of environmental uncertainty on transformational leadership and organizational resilience remains less direct and significant.

A number of studies have also revealed that organizational resilience contributes to enhancing team innovation performance by improving their adaptive capacity and collaborative innovation (Carmeli et al., 2009). Environmental uncertainty is generally associated with a high degree of uncertainty and complexity that require organizations to be flexible and responsive (Volberda, 1996). As a result, the rapid change in markets and technologies with increasing uncertainty motivates organizations and teams to continuously innovate in order to maintain their competitiveness (Tushman & O’Reilly, 1996). In this situation, organizational resilience allows teams to respond quickly and organize effectively when confronting risks, meanwhile seizing opportunities for innovation and enhancing teams’ innovative performance (Lengnick-Hall et al., 2011). Addressing environmental uncertainty requires organizations to maintain a high level of flexibility with respect to resource allocation in response to rapidly changing market demands (Eisenhardt & Martin, 2000). Whereas organizational resilience enables teams to respond to changes more effectively by triggering the optimization of resource allocation, thereby enhancing their innovation performance (Teece et al., 1997). In an environment characterized by a high degree of uncertainty, where risks and opportunities coexist (March, 1991). Enterprises with a high level of organizational resilience have superior innovative performance in the context of environmental uncertainty (Gibson & Birkinshaw, 2004). Accordingly, environmental uncertainty substantially moderates the relationship between organizational resilience and team innovation performance by increasing uncertainty, requiring optimization in resource allocation and risk management. The importance of organizational resilience becomes even more prominent in highly uncertain environments, which enables teams to cope with contextual changes and seize innovation opportunities, ultimately enhancing their innovation performance.

## 3. Research Design and Data Collection

### 3.1. Research Hypotheses

On the basis of the preceding discussion of team innovation performance, transformational leadership, organizational resilience, and environmental uncertainty, this study proposes the following hypotheses.

This study further explores the effect of transformational leadership on team innovation performance and puts forth the first hypothesis as follows:

**Hypothesis** **1.** *Transformational leadership has a significant effect upon team innovation performance.*

Regarding the impact of transformational leadership on organizational resilience and team innovation performance, the second hypothesis is proposed below:

**Hypothesis** **2.** *Transformational leadership contributes significantly to organizational resilience.*

This study proposes the third hypothesis regarding the mediating role of organizational resilience as follows:

**Hypothesis** **3.** *Organizational resilience has a mediating effect among the relationship between transformational leadership behavior and team innovation performance.*

Ultimately, this research proposes the fourth hypothesis concerning the moderating role of environmental uncertainty as follows:

**Hypothesis** **4.** *Environmental uncertainty performs a significant moderating role in moderating the relationship between organizational resilience and team innovation performance.*

### 3.2. Data Collection

**Figure 1:**
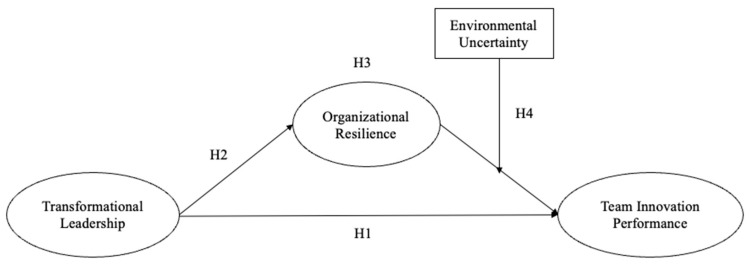
Theoretical framework and models.

Through literature review and analysis, the variables affecting team innovation performance are demonstrated in Figure 1. After conducting a field study comprising interviews with enterprise leaders, executives, middle-level and grass-roots managers, experts, and scholars in the field of economics and management, a questionnaire titled “Optimizing Team Innovation Performance in Guizhou Province in 2024” was designed. In total, 218 questionnaires were received, and after deleting the invalid samples and missing data, the number of valid samples remained 200. This research employs SPSS to analyze the collected data for regression analysis.

### 3.3. Key Variables

The dependent variable of this research is team innovation performance. Based on the Team Innovation Performance Measurement Indicators developed by Lovelace et al. (2001), consisting of four dimensions, the team’s adaptability to change, the number of innovations or new ideas introduced by the team, the team’s overall technical performance, and the innovativeness of the team’s product. The researchers of this study developed relevant questions in the questionnaire, including “The product of your organization is very innovative”; “Your organization generates a large number of innovations or new ideas”, and “The overall technical performance of your organization is very high”. The scale ranges from “strongly disagree, disagree, neutral, agree, and strongly agree”, which were assigned values from 1 to 5, respectively. The Cronbach’s alpha value of this variable is 0.922, indicating a high degree of internal consistency and reliability, which is an effective measure of team innovation performance.

The independent variable of this research is transformational leadership. The dimensions of transformational leadership were designed on the basis of the four-dimensional structure of transformational leadership demonstrated by Bass and Avolio (1996) and the localization of this structure into Chinese culture introduced by C. P. Li and Shi (2005), including moral example, visionary inspiration, charisma, and personalized care. This study recognized the similarities between moral example and charisma and therefore combined these two factors into “leadership charisma”. The specific questions measuring transformational leadership designed in the questionnaire are as follows, “The manager of your enterprise has leadership charisma”; “Your enterprise has visionary inspiration for employees”, and “Your enterprise has personalized care for employees”. A scale ranging from “very low, low, average, high, and very high” was assigned values from 1 to 5, respectively. The Cronbach’s alpha value for this variable is 0.755, which indicates a high level of internal consistency and reliability.

The mediating variable is organizational resilience. The indicators that measure organizational resilience were based on Burnard and Bhamra’s (2019) definition, which characterizes organizational resilience as the ability of an organization to withstand disruptive events and respond effectively to potential opportunities, along with the “scale of organizational resilience” demonstrated by Chinese scholars X. E. Zhang and Teng (2021). In particular, the relevant questions in the questionnaire were designed as follows: “Your enterprise continuously observes current situations in its industry in order to generate early warnings of emerging problems”; “Your enterprise is prepared to deal with emergency situations”; “Your enterprise has the ability to respond and solve problems when crises occur”; and “Your enterprise has the ability to seize new opportunities as a result of changes and crises”. The scale from “strongly disagree, disagree, neutral, agree, strongly agree”, was assigned values from 1 to 5, respectively. The Cronbach’s Alpha value of this variable is 0.782, which demonstrates a high level of internal consistency and reliability.

The moderating variable of this study is environmental uncertainty, assessed through four questions in the questionnaire, including “The number and types of products or services in the market are constantly changing”; “The technological progress in your industry is increasingly rapid”; “Your customers are constantly making new demands regarding your products or services”; and “Your enterprise has difficulty in predicting the actions taken by your competitors”. The scale of “strongly disagree, disagree, neutral, agree, strongly agree”, was assigned values from 1 to 5, respectively. The Cronbach’s alpha value of this variable is 0.940, with high internal consistency and reliability.

The control variables consisted of gender, age, educational background, enterprise type, total investment, number of employees, time of registration, location, and industry. The rationale for choosing each control variable is listed below. According to Eagly and Karau’s (2002) Role Congruity Theory, gender stereotypes can influence leadership perceptions, affecting a variety of work-related outcomes. Gender is included as a control variable to account for potential biases and differences in leadership effectiveness and employee responses. Langfred (2004) supported the inclusion of age as a control variable in studies investigating work-related attitudes and behaviors. Guo et al. (2021) examined how educational level diversity affects team creativity, highlighting the importance of considering educational background as a control variable in research regarding team performance. The specific description of each variable is presented in detail in Table 1. In addition, the KMO measure of the holistic model is 0.941, sig. = 0.000, which is greater than 0.7, resulting in a relatively high structural validity of the model.

## 4. Empirical Analysis Results

### 4.1. Descriptive Statistics

Table 2 presents the descriptive statistics of all the variables involved in this study. The correlations of the variables range from −0.363 to 0.811 without multicollinearity. The standard deviations of each variable are small, and data fluctuations remain modest and stable. From the correlation coefficients between these variables, there is a significant positive correlation between transformational leadership and team innovation performance. The direction of the correlation coefficient is generally consistent with the hypothesis, indicating its robust validity.

### 4.2. The Regression Results

The process of conducting this empirical study is summarized below. This study first examined the relationship between transformational leadership and team innovation performance. Secondly, it demonstrated the mediating role of organizational resilience between transformational leadership and team innovation performance, then detected the moderating role of environmental uncertainty between organizational resilience and team innovation performance, and finally conducted the robustness test.

Table 3 demonstrates the results of examining the relationship between transformational leadership, organizational resilience, environmental uncertainty, and team innovation performance. Model 1 is the baseline model, which contains all the control variables, namely gender, age, education, enterprise type, total investment, number of employees, registration time, location, and industry. Model 2 is the main effect model to examine the influence of the independent variable and control variables on the dependent variable. Models 3 and 4 are mediation effect analysis models. Model 3 demonstrates the effect of the independent variable on the mediating variable, and model 4 reflects the mediation effect produced by the mediating variable with respect to the independent and the dependent variables. Model 5 is a full model.

In particular, the variable transformational leadership is introduced in Model 2 to examine the effect of it on team innovation performance. The findings reveal that transformational leadership has a substantial and positive effect on team innovation performance at a level below 0.1% (beta = 0.561, *p* < 0.001), implying that the increased transformational leadership is associated with enhanced team innovation performance. The main effect test proves significant, confirming that Hypothesis 1 is valid: transformational leadership has a substantial effect on team innovation performance. Model 3 measures the relationship between transformational leadership and organizational resilience. In this case, organizational resilience is replaced as the dependent variable. Transformational leadership and control variables are also introduced. The result indicates that transformational leadership has a significant positive effect on organizational resilience (beta = 0.554, *p* < 0.001), reflecting that Hypothesis 2 is valid: the stronger the transformational leadership, the higher the team’s organizational resilience remains. Model 4 explores the mediating effect of organizational resilience among transformational leadership and team innovation performance, setting team innovation performance as the dependent variable and then introducing key variables, including transformational leadership, organizational resilience, and the control variables. The findings suggest that the coefficient of transformational leadership’s influence on team innovation performance decreases, but the direction and significance of the influence remain unchanged (beta = 0.254, *p* < 0.001). In addition, organizational resilience also has a significant positive effect on team innovation performance (beta = 0.552, *p* < 0.001). The empirical results prove that transformational leadership behaviors influence team innovation performance through organizational resilience, hence validating Hypothesis 3.

Table 4 focuses on the moderating effect mechanisms of the relationship between organizational resilience and team innovation performance. Model 1 is the baseline model containing all control and moderating variables. Models 2 and 3 test the moderating effect mechanism for organizational resilience. Specifically, Model 2 demonstrates a significant positive effect of organizational resilience on team innovation performance (beta = 0.703, *p* < 0.001). Model 3 reflects a significant negative correlation between the interaction of organizational resilience and environmental uncertainty on team innovation performance (beta = −0.447, *p* < 0.05), which supports Hypothesis 4: when the environmental uncertainty becomes more severe, the impact of organizational resilience on team innovation performance declines.

### 4.3. Robustness Test

#### Substitutability Test of Mediating Variables

To ensure the robustness of the research results, this research adopted the method of substitution measurement of key variables to test the robustness of the regression results. The mediating variable organizational resilience constituted the key variable in this research. As discussed above, the mediating variable was measured with reference to Burnard and Bhamra’s (2019) definition of organizational resilience, which is the ability of organizations to withstand and recover from disruptions and to respond to potential opportunities. In the robustness test, this research adopted a different approach to assess organizational resilience, conceiving it not as an inherent ability but as a process in which actors cultivate and apply their capabilities to engage with the environment, enabling positive adaptation and maintaining effective management of an organization throughout the early, middle, and late phases of adversity. The questions consisted of, “Your enterprise has already established early warning and coping strategies before crises occur”; “Your enterprise has the ability to quickly coordinate resources to solve problems when crises occur”; “Your enterprise can draw upon lessons learned after addressing crises”; “Your enterprise can improve comprehensively following crises”.

The results of the robustness test are presented in Table 5. Model 1 reflects that transformational leadership still has a significant positive effect on team innovation performance after introducing the control variables and the mediating variable organizational resilience with substitution (beta = 0.164, *p* < 0.05). In addition, after substituting the data, organizational resilience also has a significant positive effect on team innovation performance (beta = 0.714, *p* < 0.001). Model 2 tests the moderating effect mechanism between the mediating variable organizational resilience with substituted data and the dependent variable team innovation performance. The results of the test reflect a significant negative relationship among the interaction term between environmental uncertainty and organizational resilience in relation to team innovation performance (beta = −0.364, *p* < 0.1). The results of the alternative measures regressions of the key variables were all consistent with the hypotheses, reflecting the stability of the research findings.

## 5. Conclusions

Team innovation performance is of great significance to the economic growth, the survival, and the development of enterprises. Located in the southwest of China, the development of Guizhou Province is restricted by its economic foundation, geographic remoteness, and institutional mechanisms, which require further improvement of team innovation performance among its local enterprises. This study conducted an empirical analysis on the relationship between transformational leadership, organizational resilience, environmental uncertainty, and team innovation performance. It is discovered that transformational leadership influences team innovation performance to a great extent.

Transformational leadership contributes significantly to team innovation performance. This study examined transformational leadership in the Chinese context and proposed that it consists of three dimensions, including the leadership charisma of enterprise managers, the enterprise’s visionary incentives for employees, and the enterprise’s individualized care for employees. These three dimensions directly affect team innovation performance. Indeed, the stronger the transformational leadership, the greater the innovation of an enterprise in generating the final product, and the more creative and technologically advanced the enterprise becomes.

Organizational resilience is significantly enhanced by transformational leadership; the more robust the transformational leadership, the greater the organizational resilience. Since charismatic leadership, the enterprise’s visionary incentives and individualized care for employees trigger the enhancement of the enterprise’s organizational resilience, including having the ability to observe the situation of its industry in the long term, to provide early warnings of emerging challenges, to respond to emergency situations, and to adapt and solve problems during crises, ultimately to grasp new opportunities that arise.

Team innovation performance is influenced by transformational leadership through organizational resilience. Under the conditions of strong leadership charisma, the enterprise’s visionary motivation and individualized care for employees, enterprises are able to improve their ability to observe and identify new problems in the industry; respond to emergencies; adapt to crises; address problems; turn crises into opportunities; and ultimately strengthen team innovation performance, including the innovation and uniqueness of their product, as well as the creativity and the technical level of the enterprise.

This research argued that the stronger the environmental uncertainty, the weaker the effect of organizational resilience on team innovation performance. In general situations, organizational resilience triggers team innovation performance. However, in the context of a highly volatile and uncertain environment, the impact of organizational resilience on team innovation performance becomes weaker instead. Specifically, with the intervention of a series of factors, such as the changing number and variety of products or services in the market, the increasingly rapid technological progress in the industry, the continuous new demands made by customers regarding the enterprise’s products or services, and the difficulty of the enterprise in anticipating the actions taken by its competitors, the ability of enterprises to observe and identify new problems, tackle emergency situations, respond to crises and solve problems, and turn crises into new problems, becomes less influential on the innovativeness and distinctiveness of work results, as well as on the creative and the technological level of the enterprise. Therefore, in order to ensure the positive effect of transformational leadership and organizational resilience on the innovative performance of teams, the uncertainty of the environment ought to be subjected to appropriate control and intervention.

Based on the above discussion, this research paper proposes further suggestions for policy formulation and improvement to promote team innovation performance in various dimensions. In particular, strong transformational leadership contributes to the improvement of team innovation performance, while the development of transformational leadership requires fostering the entrepreneurial spirit of business managers. Universities and research institutes are recommended to provide additional entrepreneurship-related courses and to establish a tutoring system to encourage managers to join the studies and discussions. Meanwhile, the promotion of entrepreneurship shall be strengthened, and the case studies of outstanding entrepreneurs are encouraged to be shared. In addition, entrepreneurs from private sectors ought to be given the opportunity to participate in policy design and to be heard in economic decision-making. Private entrepreneurs are also suggested to participate in organizations such as the National People’s Congress, the Chinese People’s Political Consultative Conference, and the Federation of Industry and Commerce, to contribute to the local economic decision-making process, to share their experiences and needs from the perspective of business management, and thus assist in the optimization of economic policies.

Robust organizational resilience also promotes team innovation performance, but as explained above, elements of the environmental uncertainty diminish the impact of organizational resilience on team innovation performance. How to reduce the negative impact of environmental uncertainty turns out to be a thorny issue. Optimizing the business environment consists of providing beneficial conditions for the development of enterprises, especially private capital. Rather than merely supporting private capital through policies such as establishing funds for start-ups, providing tax incentives, and simplifying the administrative approval process, it is paramount to guarantee that private capital and state-owned capital have the opportunity to compete on a level playing field, so as to strengthen the market environment for fair competition. In addition, the government ought to promote the layout of the industrial chain, enabling enterprises to invest in and finance critical components of the chain, fostering collaborative development by supporting enhanced cooperation between upstream and downstream entities, optimizing industrial organization, and enhancing the competitiveness and resilience of enterprises in the context of an uncertain business environment. Ultimately, it is essential to strengthen support for enterprises in the financial and monetary sectors. Improving the development of enterprise credit databases guarantees that credit information is accessible, transparent, and accurate, thereby facilitating high-quality enterprises’ access to loans and other financial assistance in a clear and efficient manner. This research proposes that by optimizing the business environment, enhancing industrial chain structure, and complementing fiscal and financial support, the adverse effects of organizational resilience on team innovation performance, triggered by environmental uncertainty, are able to be effectively alleviated.

## Figures and Tables

**Table 1:** Variable description and reliability.

Variables	Name of Variable	Indicators That Measure Each Variable	Cronbach’s Alpha	Measuring the Value of Each Variable
Dependent Variable	Team innovation performance	1. The product of your enterprise is very innovative. 2. Your enterprise generates a large number of innovations or new ideas. 3. The overall technical performance of your enterprise is very high.	0.922	Strongly disagree = 1, strongly agree = 5, positively distributed.
Independent variable	Transformational leadership	1. The manager of your enterprise has leadership charisma. 2. Your enterprise has visionary inspiration for employees. 3. Your enterprise has personalized care for employees.	0.755	Very low = 1, very high = 5, positively distributed.
Mediating variable	Organizational resilience	1. Your enterprise continuously observes current situations in its industry in order to generate early warnings of emerging problems. 2. Your enterprise is prepared to deal with emergency situations. 3. Your enterprise has the ability to respond and solve problems when crises occur. 4. Your enterprise has the ability to seize new opportunities as a result of changes and crises.	0.782	Strongly disagree = 1, strongly agree = 5, positively distributed.
Moderating variable	Environmental uncertainty	1. The number and types of products or services in the market are constantly changing. 2. The technological progress in your industry is increasingly rapid. 3. Your customers are constantly making new demands regarding your products or services. 4. Your enterprise has difficulty in predicting the actions taken by your competitors.	0.940	Strongly disagree = 1, strongly agree = 5, positively distributed.
Control variables	Gender	Your gender	-	Male = 1, Female = 2.
	Age	Your age	-	20–24 = 1.51 and above = 7, positively distributed.
	Educational background	Your educational background	-	Secondary school (junior college) and below = 1, Master’s degree and above = 4, positively distributed.
	Type of Enterprise	The type of business operating in your enterprise (including ownership structure)	-	State-owned enterprises = 1, Collectively owned enterprises = 2, Private enterprises = 3, Foreign-invested enterprises = 4, Hong Kong, Macao, and Taiwan enterprises = 5.
	Total investment	Total investment in your enterprise	-	1 million (RMB) and below = 1, 50 million (RMB) and above = 5, positively distributed.
	The number of employees	The number of employees in your enterprise	-	Less than 20 employees = 1, more than 1000 employees = 5, positively distributed.
	Time of registration	Registration time of your enterprise	-	Below 1 year = 1, more than 10 years = 5, positively distributed.
	Location	Location of your enterprise (city or state)	-	Guiyang = 1, Zunyi = 2, Liupanshui = 3, Anshun = 4, Bijie = 5, Tongren = 6, Qiandongnan state = 7, Qiannan state = 8, Qianxinan state = 9, other provinces and cities = 10.
	Industry	The industry that your enterprise operates in	-	Wholesale and retail trade = 1, Accommodation and restaurants industry = 2, Information transmission, software, and information technology services = 3, Finance = 4, Rental and commercial services = 5, Education sector = 6, Health and social work sector = 7, Cultural, sports, and entertainment sector = 8, Others = 9.

**Table 2:** Means, standard deviations, and correlation coefficients of key variables.

Variables	1	2	3	4	5	6	7	8	9	10	11	12	13
1. Gender	1												
2. Age	−0.206 **	1											
3. Educational background	−0.258 ***	−0.061	1										
4. Type of Enterprise	−0.091	0.310 ***	−0.018	1									
5. Total investment	−0.154 **	0.019	0.258 ***	−0.363 ***	1								
6. The number of employees	−0.094	−0.091	0.258 ***	−0.248 ***	0.529 ***	1							
7. Time of registration	0.052	0.082	−0.125 *	−0.108	0.394 ***	0.411 ***	1						
8. Location	0.073	−0.094	−0.239 ***	−0.255 ***	0.089	−0.014	−0.070	1					
9. Industry	−0.076	−0.108	0.143 **	0.035	0.070	0.142 **	0.014	0.029	1				
10. Environmental uncertainty	−0.116	0.098	0.401 ***	0.145 **	0.110	0.082	−0.011	−0.272 ***	0.001	1			
11. Transformational leadership	−0.038	0.088	0.308 ***	0.045	0.137	0.019	−0.025	−0.174 **	0.071	0.663 ***	1		
12. Organizational resilience	−0.111	0.196 **	0.362 ***	0.088	0.171 **	0.086	0.028	−0.167 **	0.000	0.811 ***	0.629 ***	1	
13. Team innovation performance	−0.096	0.148 **	0.310 ***	0.135	0.139 **	0.110	0.000	−0.142 **	0.003	0.779 ***	0.607 ***	0.730 ***	1
Mean	1.44	3.64	2.82	1.53	3.75	3.14	3.39	3.92	6.03	2.6	2.88	2.09	2.51
ST. D	0.498	1.61	1.037	0.907	1.387	1.281	0.838	3.339	3.082	0.922	0.875	0.703	0.868

*** *p* < 0.001, ** *p* < 0.05, * *p* < 0.1.

**Table 3:** Test of the relationship between transformational leadership and team innovation performance.

Variables	Independent Variable: Team Innovation Performance		Independent Variable: Transformational Leadership	Independent Variable: Team Innovation Performance	
	Model 1	Model 2	Model 3	Model 4	Model 5
**Control variables**					
Gender	0.039 (0.544)	−0.009 (−0.147)	−0.005 (−0.088)	−0.006 (−0.118)	0.007 (0.150)
Age	0.124 * (1.675)	0.068 (1.108)	0.134 ** (2.256)	−0.006 (−0.108)	0.035 (0.717)
Educational background	0.282 *** (3.539)	0.121 * (1.773)	0.2 ** (3.047)	0.01 (0.174)	−0.025 (−0.466)
Type of enterprise	0.147 * (1.846)	0.134 ** (2.041)	0.051 (0.804)	0.106 * (1.897)	0.066 (1.274)
Total investment	0.118 (1.307)	0.028 (0.371)	0.034 (0.471)	0.009 (0.142)	0.023 (0.392)
Number of employees	0.042 (0.492)	0.108 (1.518)	0.023 (0.338)	0.095 (1.577)	0.076 (1.378)
Time of registration	−0.027 (−0.33)	−0.014 (−0.208)	0.04 (0.61)	−0.036 (−0.626)	−0.032 (−0.606)
Location	−0.039 (−0.533)	0.026 (0.418)	0.005 (0.089)	0.023 (0.437)	0.069 (1.442)
Industry	−0.039 (−0.558)	−0.069 (−1.211)	−0.061 (−1.113)	−0.035 (−0.726)	−0.015 (−0.339)
**Independent variable**					
Transformational leadership		0.561 *** (9.378)	0.554 *** (9.626)	0.254 *** (4.106)	0.511 *** (6.211)
Mediating variable					
Organizational resilience				0.552 *** (8.619)	0.132 ** (2.214)
Moderating variable					
Environmental uncertainty					0.232 ** (2.969)
Constant	1.122	0.16	0.029	0.14	0.009
Pseudo R^2^	0.108	0.388	0.432	0.559	0.632
Observations	200	200	200	200	200

*** *p* < 0.001, ** *p* < 0.05, * *p* < 0.1, *t*-values in brackets.

**Table 4:** Test of moderating effect of the relationship between organizational resilience and team innovation performance.

Variables	Independent Variable: Team Innovation Performance		
	Model 1	Model 2	Model 3
**Control variables**			
Gender	0.022 (0.465)	0.009 (0.177)	0.015 (0.317)
Age	0.075 (1.520)	−0.009 (−0.162)	0.05 (1.023)
Educational background	−0.002 (−0.034)	0.029 (0.474)	−0.003 (−0.058)
Type of enterprise	0.05 (0.933)	0.102 * (1.755)	0.063 (1.217)
Total investment	0.049 (0.811)	0.031 (0.472)	0.027 (0.471)
Number of employees	0.055 (0.972)	0.072 (1.145)	0.055 (0.995)
Registration time	−0.029 (−0.542)	−0.046 (−0.768)	−0.035 (−0.661)
Location	0.082 * (1.658)	0.002 (0.041)	0.062 (1.291)
Industry	−0.003 (−0.058)	−0.017 (−0.332)	−0.002 (−0.049)
**Mediating variable**			
Organization resilience		0.703 *** (12.868)	0.506 *** (3.765)
Moderating variable			
Environmental uncertainty	0.780 *** (15.448)		0.779 *** (6.333)
Organization resilience × environmental uncertainty			−0.447 ** (−2.188)
Constant	0.120	0.428	−0.399
Pseudo R^2^	0.604	0.522	0.632
Observations	200	200	200

*** *p* < 0.001, ** *p* < 0.05, * *p* < 0.1.

**Table 5:** Robustness test.

Variables	Independent Variable: Team Innovation Performance	
	Model 1	Model 2
Control variables		
Gender	0.001 (0.033)	0.011 (0.265)
Age	0.002 (0.041)	0.027 (0.591)
Educational background	−0.026 (−0.508)	−0.022 (−0.422)
Type of enterprise	0.032 (0.659)	0.034 (0.709)
Total investment	0.003 (0.062)	0.021 (0.394)
Number of employees	0.107 ** (2.034)	0.073 (1.413)
Registration time	−0.081 (−1.613)	−0.072 (−1.466)
Location	0.022 (0.479)	0.042 (0.931)
Industry	−0.031 (−0.74)	−0.013 (−0.314)
**Independent variable**		
Transformational leadership	0.164 ** (3.019)	
Mediating variable		
Organizational resilience	0.714 *** (12.598)	0.717 *** (5.78)
**Moderating variable**		
Environmental uncertainty		0.515 *** (4.015)
Organization resilience × environmental uncertainty		−0.364 * (−1.854)
Constant	0.396	−0.029
Pseudo R^2^	0.666	0.683
Observations	200	200

*** *p* < 0.001, ** *p* < 0.05, * *p* < 0.1.

## Data Availability

The data used to support the findings of this study are available from the corresponding author upon request.

## References

[B1-behavsci-15-00010] Adner R. (2006). Match your innovation strategy to your innovation ecosystem. Harvard Business Review.

[B2-behavsci-15-00010] Ancona D. G., Caldwell D. F. (1992). Demography and design: Predictors of new product team performance. Organization Science.

[B3-behavsci-15-00010] Bass B. M., Avolio B. J. (1990). Developing transformational leadership: 1992 and beyond. Journal of European Industrial Training.

[B4-behavsci-15-00010] Bass B. M., Avolio B. J. (1996). Multifactor leadership questionnaire.

[B5-behavsci-15-00010] Birkinshaw J., Zimmermann A., Raisch S. (2016). How do firms adapt to discontinuous change? Bridging the dynamic capabilities and ambidexterity perspectives. California Management Review.

[B6-behavsci-15-00010] Burnard K. J., Bhamra R. (2019). Challenges for organisational resilience. Continuity & Resilience Review.

[B7-behavsci-15-00010] Burns J. M. (1978). Leadership.

[B8-behavsci-15-00010] Carmeli A., Brueller D., Dutton J. E. (2009). Learning behaviours in the workplace: The role of high-quality interpersonal relationships and psychological safety. Systems Research and Behavioral Science: The Official Journal of the International Federation for Systems Research.

[B9-behavsci-15-00010] Caza A., Caza B. B., Posner B. Z. (2021). Transformational leadership across cultures: Follower perception and satisfaction. Administrative Sciences.

[B10-behavsci-15-00010] Chen C., Yang Z. W., Wang B. B. (2023). Resilient leadership: The multi-dimensional construction, measurement, and verification the impact on organizational resilience. Journal of Management Science.

[B11-behavsci-15-00010] Chen J., Wang X., Shen W., Tan Y., Matac L. M., Samad S. (2022). Environmental uncertainty, environmental regulation and enterprises’ green technological innovation. International Journal of Environmental Research and Public Health.

[B12-behavsci-15-00010] Chen R., Xie Y., Liu Y. (2021). Defining, conceptualizing, and measuring organizational resilience: A multiple case study. Sustainability.

[B13-behavsci-15-00010] Chi D. M., Duan S. S., Zhang Y. M. (2023). Power of harmony: The impact of labor relation climate on organizational resilience. Foreign Economics & Management.

[B14-behavsci-15-00010] Choi H., Varian H. (2012). Predicting the present with Google Trends. Economic Record.

[B15-behavsci-15-00010] Davenport T. H., Ronanki R. (2018). Artificial intelligence for the real world. Harvard Business Review.

[B16-behavsci-15-00010] Deng X. M., Liu Y., Long X. Y., Lin X. Z., Yang S. Y. (2021). A Study on the relationship between environmental dynamism and strategic change based on the perspective of managerial cognition. Nankai Business Review.

[B17-behavsci-15-00010] Dess G. G., Beard D. W. (1984). Dimensions of organizational task environments. Administrative Science Quarterly.

[B18-behavsci-15-00010] Downton J. V. (1973). Rebel leadership: Commitment and charisma in the revolutionary process.

[B19-behavsci-15-00010] Duncan R. B. (1972). Characteristics of organizational environments and perceived environmental uncertainty. Administrative Science Quarterly.

[B20-behavsci-15-00010] Eagly A. H., Karau S. J. (2002). Role Congruity Theory of Prejudice Toward Female Leaders. Psychological Review.

[B21-behavsci-15-00010] Eisenhardt K. M. (1989). Making fast strategic decisions in high-velocity environments. Academy of Management Journal.

[B22-behavsci-15-00010] Eisenhardt K. M., Martin J. A. (2000). Dynamic capabilities: What are they?. Strategic Management Journal.

[B23-behavsci-15-00010] Farh L. J. L., Cheng B. S., Chou L. F. (2000). A triad model of paternalistic leadership: Constructs and measurement. Indigenous psychological research in Chinese societies.

[B24-behavsci-15-00010] Farzana S., Charoensukmongkol P. (2024). Effects of transformational leadership on psychological empowerment and employee engagement: A comparative study of Bangladesh and Thailand. Journal of Asia Business Studies.

[B25-behavsci-15-00010] Gao Z. L., Tan W. H., Mao L. X. (2024). Organizational resilience and the innovation quality of SRDI small and medium-sized enterprises. Science & Technology Progress and Policy.

[B26-behavsci-15-00010] Gibson C. B., Birkinshaw J. (2004). The antecedents, consequences, and mediating role of organizational ambidexterity. Academy of Management Journal.

[B27-behavsci-15-00010] Guo W., Gan C., Wang D. (2021). When does educational level diversity foster team creativity? Exploring the moderating roles of task and personnel variability. Frontiers in Psychology.

[B28-behavsci-15-00010] Guo W., Li Y. P., Du J., Tao H. Y. (2012). Authentic leadership and employee-team creativity: A multi-level Study. Nankai Business Review.

[B29-behavsci-15-00010] Heredia J., Castillo-Vergara M., Geldes C., Gamarra F. M. C., Flores A., Heredia W. (2022). How do digital capabilities affect firm performance? The mediating role of technological capabilities in the “new normal”. Journal of Innovation & Knowledge.

[B30-behavsci-15-00010] Hillmann J. (2021). Disciplines of organizational resilience: Contributions, critiques, and future research avenues. Review of Managerial Science.

[B31-behavsci-15-00010] Hou G. W., Liu Q. Q., Liu Q. L. (2023). Inverted U-shaped relationship between dual value chain embedding and innovation performance: The mediating role of enterprise digitization and adjustment of organizational resilience. Science & Technology Progress and Policy.

[B32-behavsci-15-00010] Hu D. M., Zhao L., Chen W. Z. (2021). A study on the mechanism and effect of heterogeneous characteristics of executive teams of listed companies on organizational resilience. Social Science Research.

[B33-behavsci-15-00010] Jung D. I., Chow C., Wu A. (2003). The role of transformational leadership in enhancing organizational innovation: Hypotheses and some preliminary findings. The Leadership Quarterly.

[B34-behavsci-15-00010] Kasımoğlu M., Ammari D. (2020). Transformational leadership and employee creativity across cultures. Administrative Sciences.

[B35-behavsci-15-00010] Katz R., Allen T. J. (1985). Project performance and the locus of influence in the R&D matrix. Academy of Management Journal.

[B36-behavsci-15-00010] Ke J. L., Sun J. M., Shi J. T. (2009). The effect of transformational leadership on innovation performance of R&D team. Nankai Business Review.

[B37-behavsci-15-00010] Kuntz J. R. C., Malinen S., Näswall K. (2017). Employee resilience: Directions for resilience development. Consulting Psychology Journal: Practice and Research.

[B38-behavsci-15-00010] Langfred C. W. (2004). Too much of a good thing? Negative effects of high trust and individual autonomy in self-managing teams. Academy of Management Journal.

[B39-behavsci-15-00010] Lengnick-Hall C. A., Beck T. E., Lengnick-Hall M. L. (2011). Developing a capacity for organizational resilience through strategic human resource management. Human Resource Management Review.

[B40-behavsci-15-00010] Li C. P., Shi K. (2005). The structure and measurement of transformational leadership in China. Acta Psychologica Sinica.

[B41-behavsci-15-00010] Li D., Liu J. (2014). Dynamic capabilities, environmental dynamism, and competitive advantage: Evidence from China. Journal of Business Research.

[B42-behavsci-15-00010] Li M., Zhou Y. X., Du C. P. (2024). How does platform leadership improve team innovation performance? A chain mediation analysis based on SEM and fsQCA. Science & Technology Progress and Policy.

[B43-behavsci-15-00010] Li Y., Liang L. (2024). The impact of organizational resilience: Review and prospect. East China Economic Management.

[B44-behavsci-15-00010] Liang F., Cao L. (2021). Linking employee resilience with organizational resilience: The roles of coping mechanism and managerial resilience. Psychology Research and Behavior Management.

[B45-behavsci-15-00010] Liang F., Qiu Y. X., Li Q. (2023). How platform leadership promotes the sustainable innovation capability of an organization in an ambidextrous environment: The mediating role of organizational resilience. Science & Technology Progress and Policy.

[B46-behavsci-15-00010] Lin H. F., Sun X. H. (2024). A multi-case study on the activation processes of TMT faultlines in the context of organizational change. Chinese Journal of Management.

[B47-behavsci-15-00010] Lin J. B., Fan Y. C. (2024). Seeking sustainable performance through organizational resilience: Examining the role of supply chain integration and digital technology usage. Technological Forecasting and Social Change.

[B48-behavsci-15-00010] Liu B. N., Zhang Z. X. (2023). What enables teams to “bounce back” from adversity? The influence of leader mindfulness on team resilience. Advances in Psychological Science.

[B49-behavsci-15-00010] Liu L. B., Zhang L. R., Zhao X. (2023). Research on a new measurement method of external environmental uncertainty-based on the perspective of business forecasting. Friends of Accounting.

[B50-behavsci-15-00010] Liu X. Y., Liu J. (2012). The influence mechanism of team emotional climate on team innovation performance. Acta Psychologica Sinica.

[B51-behavsci-15-00010] Lovelace K., Shapiro D. L., Weingart L. R. (2001). Maximizing cross-functional new product teams’ innovativeness and constraint adherence: A conflict communications perspective. Academy of Management Journal.

[B52-behavsci-15-00010] Ma X. F., Zhong G. Y., Rui Z. Y. (2024). Transformational leadership and transactional leadership—A comparative study on employee psychological empowerment and creativity in entrepreneurial hostile context. Soft Science.

[B53-behavsci-15-00010] March J. G. (1991). Exploration and exploitation in organizational learning. Organization Science.

[B54-behavsci-15-00010] Miller D., Friesen P. H. (1983). Strategy-making and environment: The third link. Strategic Management Journal.

[B55-behavsci-15-00010] Milliken F. J. (1987). Three types of perceived uncertainty about the environment: State, effect, and response uncertainty. Academy of Management Review.

[B56-behavsci-15-00010] Munoz A., Billsberry J. (2024). Resilience, robustness, and antifragility: Towards an appreciation of distinct organizational responses to adversity. International Journal of Materials Research.

[B57-behavsci-15-00010] Qi Z. J., Zhang X. L. (2001). Theories of Leadership Behavior: Exchange and Transformational Leadership Behavior. Enterprise Operation & Management.

[B58-behavsci-15-00010] Schilling M. A. (2017). Strategic management of technological innovation.

[B59-behavsci-15-00010] Seo E. H., Kim C. Y., Kim K. (2020). A study on the mechanisms linking environmental dynamism to innovation performance. Sustainability.

[B60-behavsci-15-00010] Sharfman M. P., Dean J. W. (1991). Conceptualizing and measuring the organizational environment: A multidimensional approach. Journal of Management.

[B61-behavsci-15-00010] Sun H. P., Hansen H. O. (2020). A study of the impact of leadership style on organizational innovation: Based on the mediating role of human resource management practices. Journal of Guangxi University (Philosophy and Social Sciences).

[B62-behavsci-15-00010] Sun M. X., Zhu F. W., Guo J. N., Guan Y. (2021). Influence of transformational leadership on team resilience: A perspective of sensemaking. Journal of Management Science.

[B63-behavsci-15-00010] Sun Y. N., Jiang L. Y. (2024). How ambidextrous innovation enhances organizational resilience: A multi-case study in the chinese context. Social Sciences in Nanjing.

[B64-behavsci-15-00010] Tang C. Y., Shi Y. Z., Li Y. B., Chen W. M. (2023). Learning from failures and firm performance: The role of organizational resilience and environmental dynamics. Management Review.

[B65-behavsci-15-00010] Tang Y. H., Zhao C. H., Jiang Z. X. (2020). The Influence Mechanism of Leader-member Exchange Differentiation on Team Innovation Performance—The Role of Team Member Exchange and Team Political Climate. Collected Essays on Finance and Economics.

[B66-behavsci-15-00010] Tao Y., He J. X., Liu H., Zhang Z. Y. (2024). Strategy configurations and competitive advantage, organizational resilience: Research based on Chinese manufacturing firms. Journal of Industrial Engineering and Engineering Management.

[B67-behavsci-15-00010] Tatikonda M. V., Rosenthal S. R. (2000). Successful execution of product development projects: Balancing firmness and flexibility in the innovation process. Journal of Operations Management.

[B68-behavsci-15-00010] Teece D. J. (2007). Explicating dynamic capabilities: The nature and micro foundations of (sustainable) enterprise performance. Strategic Management Journal.

[B69-behavsci-15-00010] Teece D. J., Pisano G., Shuen A. (1997). Dynamic capabilities and strategic management. Strategic Management Journal.

[B70-behavsci-15-00010] Tushman M. L., O’Reilly C. A. (1996). Ambidextrous organizations: Managing evolutionary and revolutionary change. California Management Review.

[B71-behavsci-15-00010] Volberda H. W. (1996). Toward the flexible form: How to remain vital in hypercompetitive environments. Organization Science.

[B72-behavsci-15-00010] Waldman D. A., Yammarino F. J. (1999). CEO charismatic leadership: Levels-of-management and levels-of-analysis effects. Academy of Management Review.

[B73-behavsci-15-00010] Wamba S. F., Queiroz M. M., Trinchera L. (2024). The role of artificial intelligence-enabled dynamic capability on environmental performance: The mediation effect of a data-driven culture in France and the USA. International Journal of Production Economics.

[B74-behavsci-15-00010] Wang C. L., Senaratne C., Rafiq M. (2015). Success traps, dynamic capabilities and firm performance. British Journal of Management.

[B75-behavsci-15-00010] Wang G. H., Lin H., Huang H. (2024a). The effect of resource orchestration on SMEs organizational resilience from the perspective of digitalization. Science & Technology Progress and Policy.

[B76-behavsci-15-00010] Wang H. H., Li Y. J., Gong Y. Y. (2024b). Technological diversification, continuous innovation and enterprise resilience: The moderating effects of a dual network. Science & Technology Progress and Policy.

[B77-behavsci-15-00010] Wang J. R., Liu J. Y. (2024). Research on the impact of boundary-spanning search and organizational resilience on green technological innovation of firms. Science Research Management.

[B78-behavsci-15-00010] Wang J. R., Liu X. Y., Ren X. H. (2024c). The impact of ambidextrous learning and organizational resilience on firms’ technological innovation. Science and Technology Management Research.

[B79-behavsci-15-00010] Wang J. R., Zhang Y. M., Fan W. Y. (2024d). Proactive boundary-spanning search, organizational resilience and ambidextrous innovation. Studies in Science of Science.

[B80-behavsci-15-00010] Wang Q., Wang Z. X., Liu Y. Q. (2023). Mechanisms through which digital transformation enhances enterprise organizational resilience. Journal of Management Sciences in China.

[B81-behavsci-15-00010] Wang Y., Cai J. (2021). Influence mechanism of positive leadership on organizational resilience. Journal of Capital University of Economics and Business.

[B82-behavsci-15-00010] Williams A., Whiteman G., Kennedy S. (2021). Cross-scale systemic resilience: Implications for organization studies. Business & Society.

[B83-behavsci-15-00010] Wu X. B., Feng X. Y. (2022). Impact of operational slack on organizational resilience in the VUCA context: The mediating role of sustainable innovation capability. Journal of Systems & Management.

[B84-behavsci-15-00010] Xin X., Miao R. T. (2018). The influential mechanism of employees’ job crafting on creative performance: A moderated double-mediation model. Economic Management Journal.

[B85-behavsci-15-00010] Ye J., Zhang X. F., Zhou L. L. (2022). Relationship between organizational resilience and innovation: A systematic review and meta-analysis. Science and Technology Management Research.

[B86-behavsci-15-00010] Yin J. L., Jia M. X., Liao G. L. (2020). Research on the impact of team leader’s conflict management styles on team innovation performance: The mediating role of team positive emotional climate. Journal of Technology Economics.

[B87-behavsci-15-00010] Yoo S., Jeong S., Song J. H., Bae S. (2020). Transformational leadership and knowledge creation practices in Korean and US schools: Knowledge assets as mediators. Knowledge Management Research & Practice.

[B88-behavsci-15-00010] Zeng F. E., Wang K. W., Chi Y. J., Xia J. (2016). The research about effects of leadership style on normal performances and innovation performances of team and its mechanism—Based on the mediation of team execution and organization identification. Science & Technology Progress and Policy.

[B89-behavsci-15-00010] Zhang B. J., Pei M. D. (2020). Dual choice of firm resilience strategies—Based on the theory of sustainable innovation. Modern Economic Research.

[B90-behavsci-15-00010] Zhang H. L., Song M., He H. H. (2021a). Solution to the “innovation speed dilemma”: A cross-national study of the roles of transformational leadership. Journal of Industrial Engineering.

[B91-behavsci-15-00010] Zhang J., Wang G. H. (2020). An analysis of the mechanisms of transformational leadership’s impact on team innovation performance. Enterprise Economy.

[B92-behavsci-15-00010] Zhang J., Zheng W. B., Zhao N. (2021b). Research on the multi level influence mechanism of leader mindfulness on organizational members’ resilience. Human Resources Development of China.

[B93-behavsci-15-00010] Zhang S. S., Dong X. D. (2024). Intelligent transformation, organizational resilience, and high-quality development of manufacturing enterprises. China Business and Market.

[B94-behavsci-15-00010] Zhang X. E., Teng X. Y. (2021). The connotation, dimensions, and measurement of organizational resilience. Science & Technology Progress and Policy.

[B95-behavsci-15-00010] Zhang Y., Hou L. W. (2013). A study on relationship between functional diversity and team knowledge sharing: The moderating role of transformational leadership. Chinese Journal of Management.

[B96-behavsci-15-00010] Zhao S. J., Yi L. F., Lian Y. L. (2021). Entrepreneurial leadership, organizational resilience and new venture performance. Foreign Economics & Management.

[B97-behavsci-15-00010] Zhou W., Li H., Zong J. N. (2024). The effect of corporate strategic changes on organizational resilience and its boundary. Science Research Management.

[B98-behavsci-15-00010] Zhu S. Y., Qi E. S., Xu Y. (2008). An empirical study of the relationship among transformational leadership style, team climate, knowledge sharing and performance of team innovation. Soft Science.

[B99-behavsci-15-00010] Zhu Y. H., Zhao Y. L., Zhou Y. Y., Wu J. (2019). Resilience in organizations: Construction of protective resources from psychological and systematic perspective. Advances in Psychological Science.

